# Delayed Diagnosis of Primary Bronchial Adenoid Cystic Carcinoma Presenting as Central Airway Obstruction: A Case Report

**DOI:** 10.7759/cureus.105295

**Published:** 2026-03-16

**Authors:** Oussama FIKRI, Kaoutar Lhachimi, Chaynez Rachid, Mohamed IJIM, Lamyae Amro

**Affiliations:** 1 Department of Pulmonology, Faculty of Medicine and Pharmacy of Marrakech (FMPM), Arrazi Hospital, Mohammed VI University Hospital Centre (CHU Mohammed VI), Cadi Ayyad University (UCA), Marrakesh, MAR; 2 Department of Pulmonology, Faculty of Medicine and Pharmacy of Marrakech (FMPM), Arrazi Hospital, Mohammed VI University Hospital Centre (CHU Mohammed VI), Cadi Ayyad University (UCA),, Marrakesh, MAR

**Keywords:** bronchial adenoid cystic carcinoma, central airway obstruction, immunohistochemistry, pneumonectomy, rare lung cancer

## Abstract

Bronchial adenoid cystic carcinoma (ACC) is a rare malignant tumor originating from salivary gland-type tissues of the tracheobronchial tree. It usually occurs in relatively young patients and is not strongly associated with tobacco exposure. Despite its slow-growing nature, ACC can behave aggressively locally, leading to significant airway obstruction.

We report the case of a 47-year-old woman exposed to wood smoke who presented with progressive exertional dyspnea and unquantified weight loss. Chest computed tomography revealed a right hilar infiltrative lesion completely obstructing the right main bronchus, causing right lung atelectasis. Bronchoscopic evaluation demonstrated a highly vascularized endobronchial tumor. Histopathological examination confirmed ACC with cribriform pattern and perineural invasion. Pulmonary function tests indicated suitability for surgery. Considering the anatomical extent and absence of distant metastasis, the patient underwent right pneumonectomy with carinal resection.

This case emphasizes the diagnostic challenges of ACC, the rationale for radical surgery in anatomically constrained tumors, and the importance of long-term multidisciplinary follow-up due to the risk of late local recurrence and delayed metastasis. Early recognition of persistent central airway symptoms is critical for optimizing patient outcomes.

## Introduction

Bronchial adenoid cystic carcinoma (ACC) is a rare malignant tumor arising from the submucosal glandular structures of the tracheobronchial tree. This tumor was first described by Theodor Billroth in 1856 [1]. ACC is considered an uncommon entity, accounting for approximately 0.1%-0.2% of primary lung tumors.

Historically, ACC was classified as a tumor of intermediate malignant potential; however, it is now recognized as a malignant neoplasm due to its locally invasive behavior, characterized by perineural invasion, slow but persistent growth, and the possibility of late regional or distant metastasis even after apparently complete surgical resection [2].

Unlike most primary lung cancers, ACC tends to occur in relatively younger patients and shows no strong association with tobacco exposure. The clinical presentation is usually insidious and dominated by symptoms related to central airway obstruction, including progressive dyspnea, cough, wheezing, stridor, or hemoptysis. Because these manifestations are nonspecific and may mimic common obstructive airway diseases such as asthma or chronic bronchitis, diagnosis is frequently delayed [2].

Early recognition of this tumor is essential, as therapeutic strategy and prognosis depend largely on tumor extent, presence of metastasis, and feasibility of complete surgical resection. Multidisciplinary management is therefore recommended for optimal patient outcomes [3].

Because of its rarity and nonspecific presentation, bronchial ACC may be overlooked during the initial diagnostic evaluation. Reporting additional cases contributes to a better understanding of their clinical presentation, diagnostic challenges, and therapeutic strategies. We report a case of primary bronchial ACC presenting with progressive airway obstruction requiring surgical management.

## Case presentation

The patient was a 47-year-old woman with a history of chronic wood-smoke exposure but no history of smoking or tuberculosis. She reported progressive exertional dyspnea evolving over approximately one year, corresponding to grade II on the Modified Medical Research Council (mMRC) scale [4], gradually worsening in intensity and limiting her daily activities. She also noted unquantified weight loss over the same period. These symptoms were directly related to progressive obstruction of the right main bronchus by the tumor, illustrating the insidious nature and delayed presentation typical of airway ACC. 

Physical examination revealed a good general condition (performance status 1). Respiratory rate was 20 breaths per minute, and oxygen saturation was 95% on room air. Chest examination demonstrated consolidation of the entire right hemithorax. No peripheral lymphadenopathy was detected.

**Figure 1:**
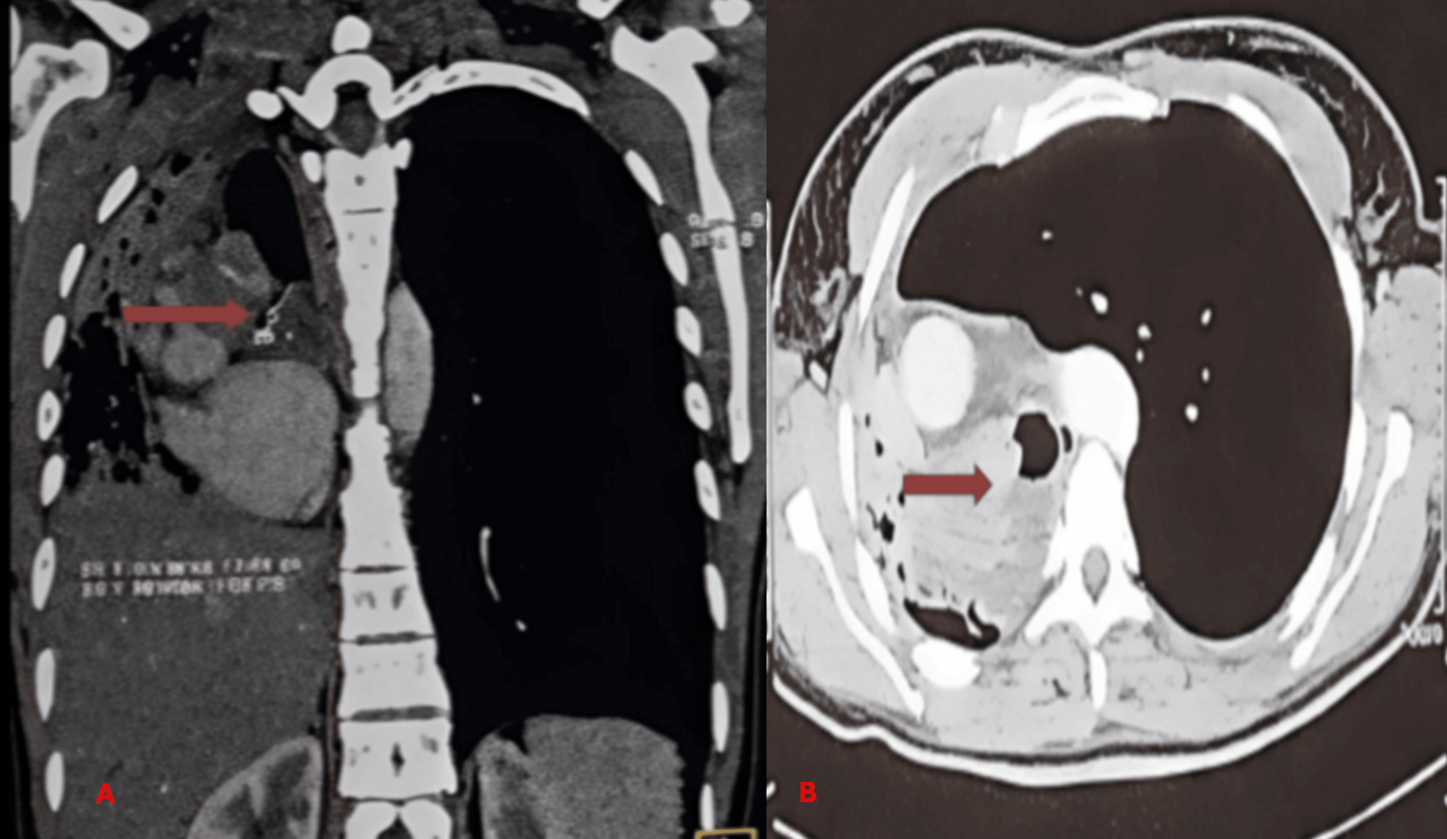
Right hilar lesion on thoracic computed tomography (CT) scan. (A) Coronal thoracic CT image showing a right hilar infiltrative lesion with heterogeneous contrast enhancement (red arrow). (B) Axial CT section confirming the right hilar mass causing bronchial obstruction (red arrow).

Thoracic computed tomography demonstrated a poorly defined right hilar infiltrative lesion measuring approximately 4 × 3 × 3 cm, involving the proximal right main bronchus and extending toward the carina up to 2 cm from it. The tumor caused complete bronchial obstruction and post-obstructive atelectasis. No clear invasion of adjacent mediastinal structures. These findings were critical in determining the feasibility of complete surgical resection and the decision to perform pneumonectomy with carinal resection (Figures 1A, 1B).

**Figure 2:**
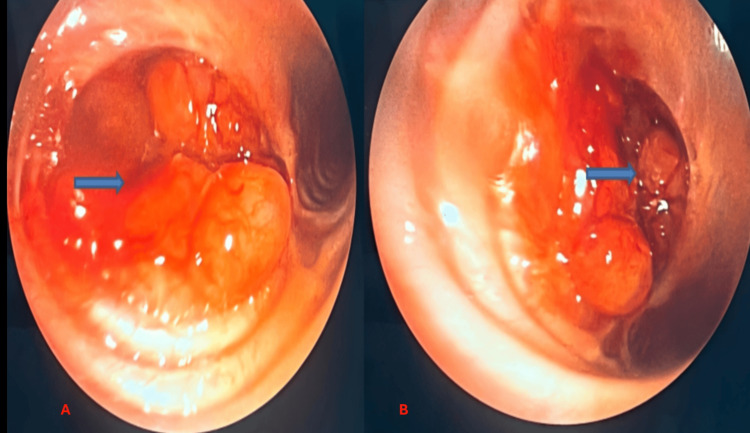
Endobronchial tumor obstructing the right main bronchus. (A) Bronchoscopic view showing a large, shiny, highly vascularized endobronchial tumor (blue arrow) occupying the right main bronchus. (B) Close-up bronchoscopic image demonstrating the tumor (blue arrow) completely occluding the bronchial lumen.

Bronchoscopic evaluation revealed a large, shiny, highly vascularized endobronchial tumor completely occluding the right main bronchus. The lesion bled on contact and prevented catheterization of the airway (Figures 2A, 2B).

**Figure 3:**
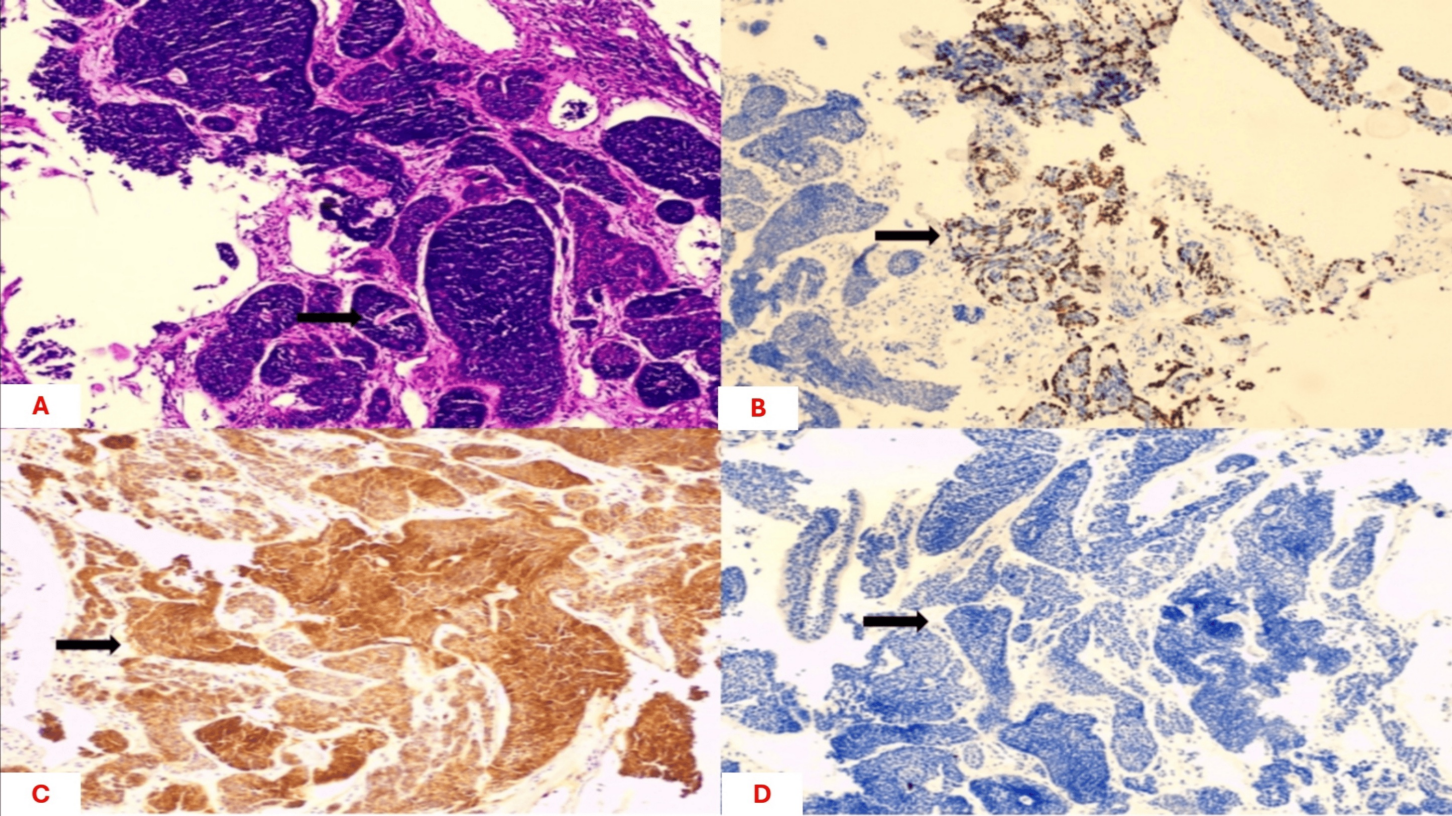
Histopathological examination of a bronchial tumor biopsy. (A) Hematoxylin and eosin staining demonstrating characteristic cribriform architecture (arrow), consistent with adenoid cystic carcinoma. (B) Immunohistochemical staining showing CD117 positivity (arrow) in tumor cells. (C) Immunohistochemical staining showing CK7 positivity (arrow) in tumor cells. (D) Immunohistochemical staining showing tumor cells negative for TTF-1 (arrow).

Histopathological examination revealed a cribriform growth pattern with characteristic perineural invasion, composed of epithelial and myoepithelial cells forming pseudocystic spaces filled with basophilic material. Immunohistochemistry was positive for p40, p63, CD117, CK5/6, and CK7, and negative for TTF-1, CK20, GATA-3, chromogranin, and synaptophysin (Figures 3A-3D). The cribriform pattern with perineural invasion is associated with an intermediate prognostic profile and a risk of late local recurrence, which guided our postoperative follow-up strategy (Figures 3A-3D).

**Figure 4:**
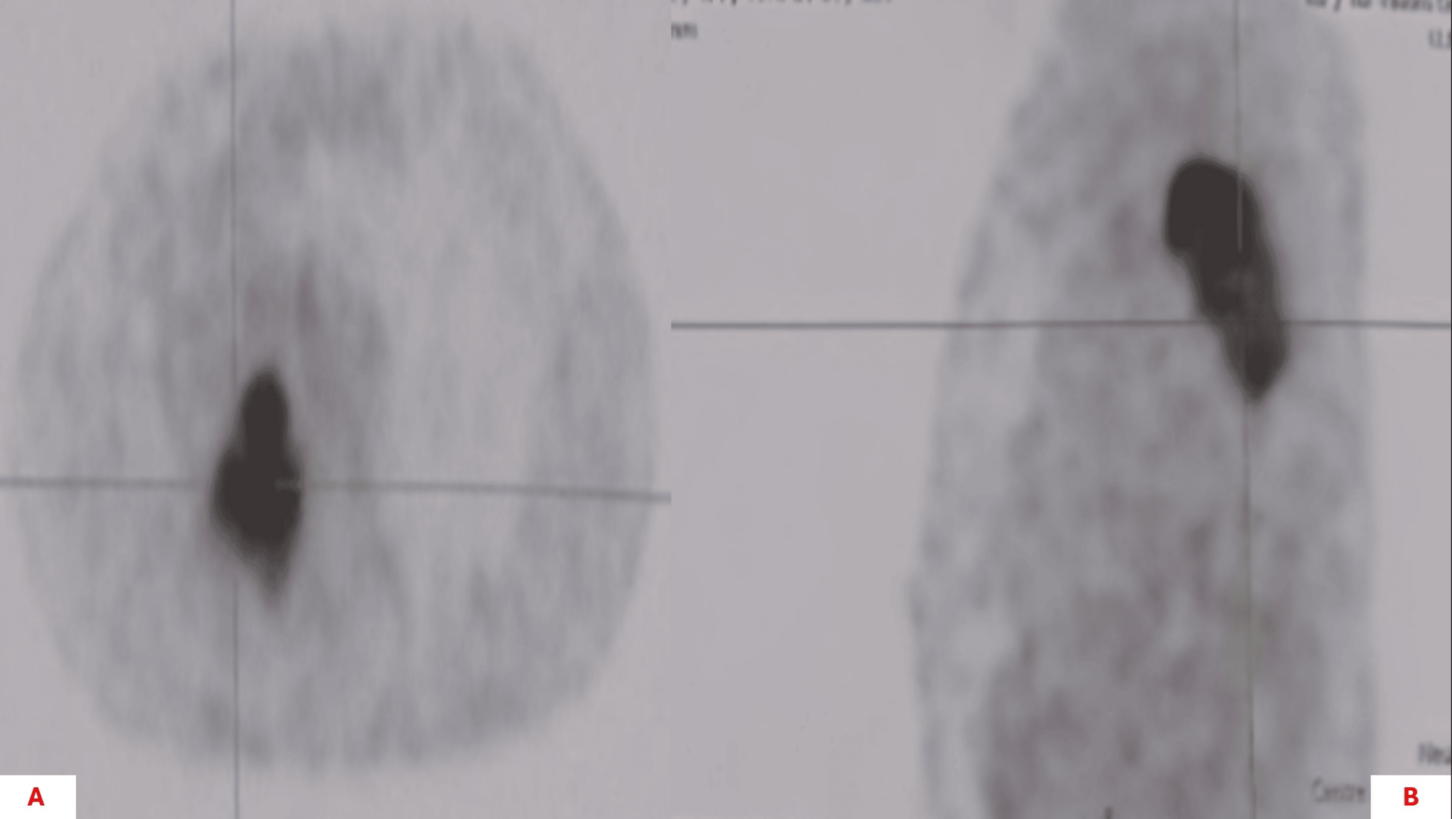
Positron emission tomography (PET) imaging. (A) Axial PET fusion image showing increased metabolic uptake in the right hilar lesion. (B) Coronal whole-body PET image demonstrating focal hypermetabolism in the right hilum.

Positron emission tomography (PET) demonstrated a metabolically active right hilar lesion without evidence of distant metastasis (Figures 4A, 4B).

Pulmonary function tests were performed as part of the preoperative assessment and demonstrated preserved respiratory function compatible with surgical management.

**Figure 5:**
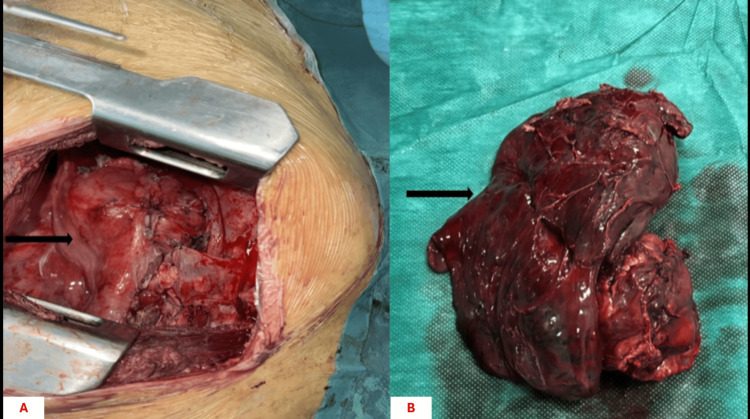
Surgical management by right pneumonectomy with carinal resection. (A) Intraoperative view illustrating right pneumonectomy with carinal resection performed for complete bronchial obstruction caused by the tumor (arrow). (B) Surgical specimen of the resected lung containing the bronchial tumor (arrow).

Given the complete obstruction of the right main bronchus and the extent of bronchial involvement, lung-preserving procedures were considered technically unfeasible. Therefore, right pneumonectomy with carinal resection was performed to achieve complete tumor removal with negative surgical margins (Figures 5A, 5B).

**Figure 6:**
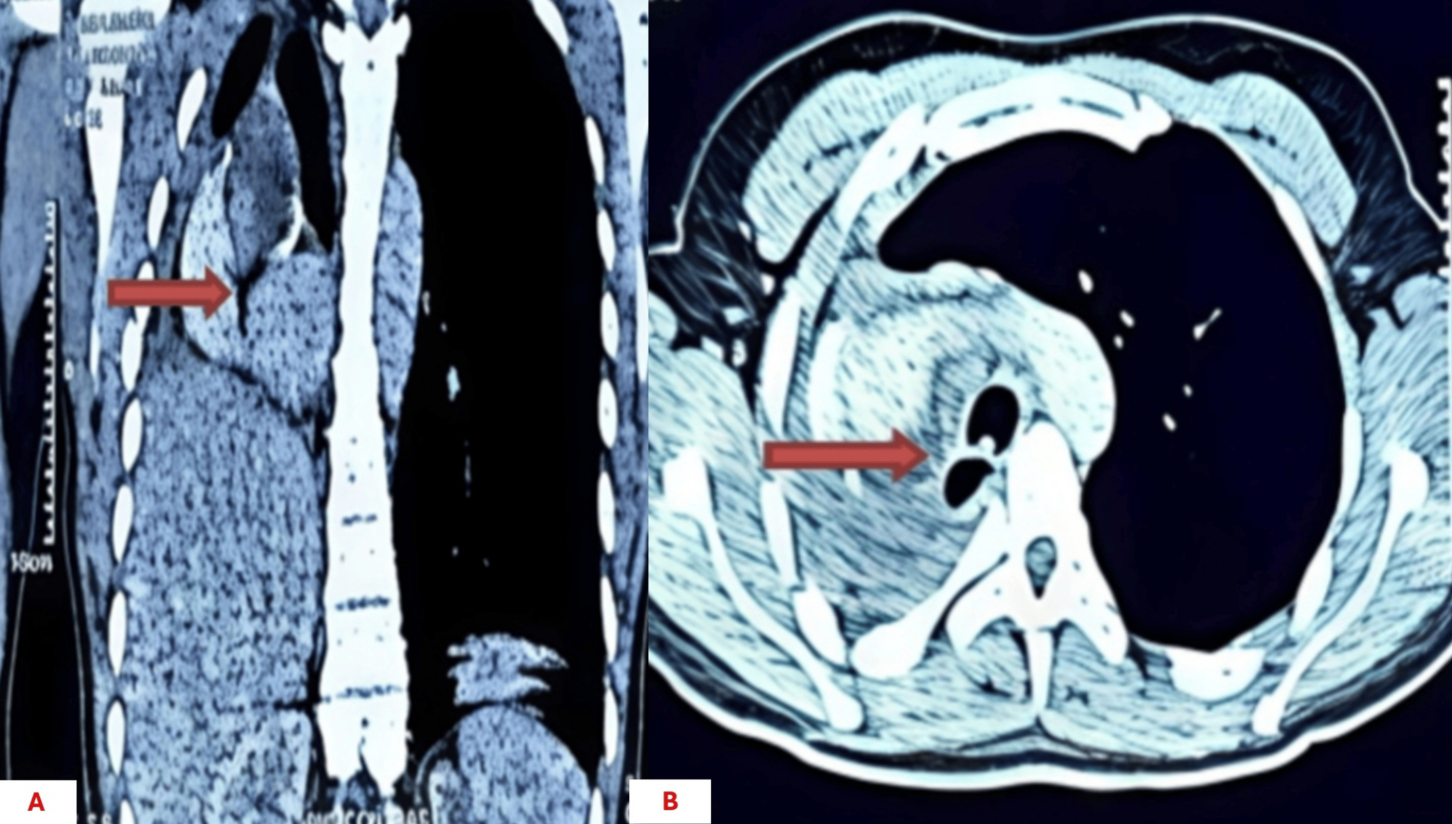
Postoperative thoracic CT scan. (A) Coronal CT image showing postoperative changes (red arrow) following right pneumonectomy. (B) Axial CT image demonstrating mediastinal shift and absence of residual tumor (red arrow).

**Figure 7:**
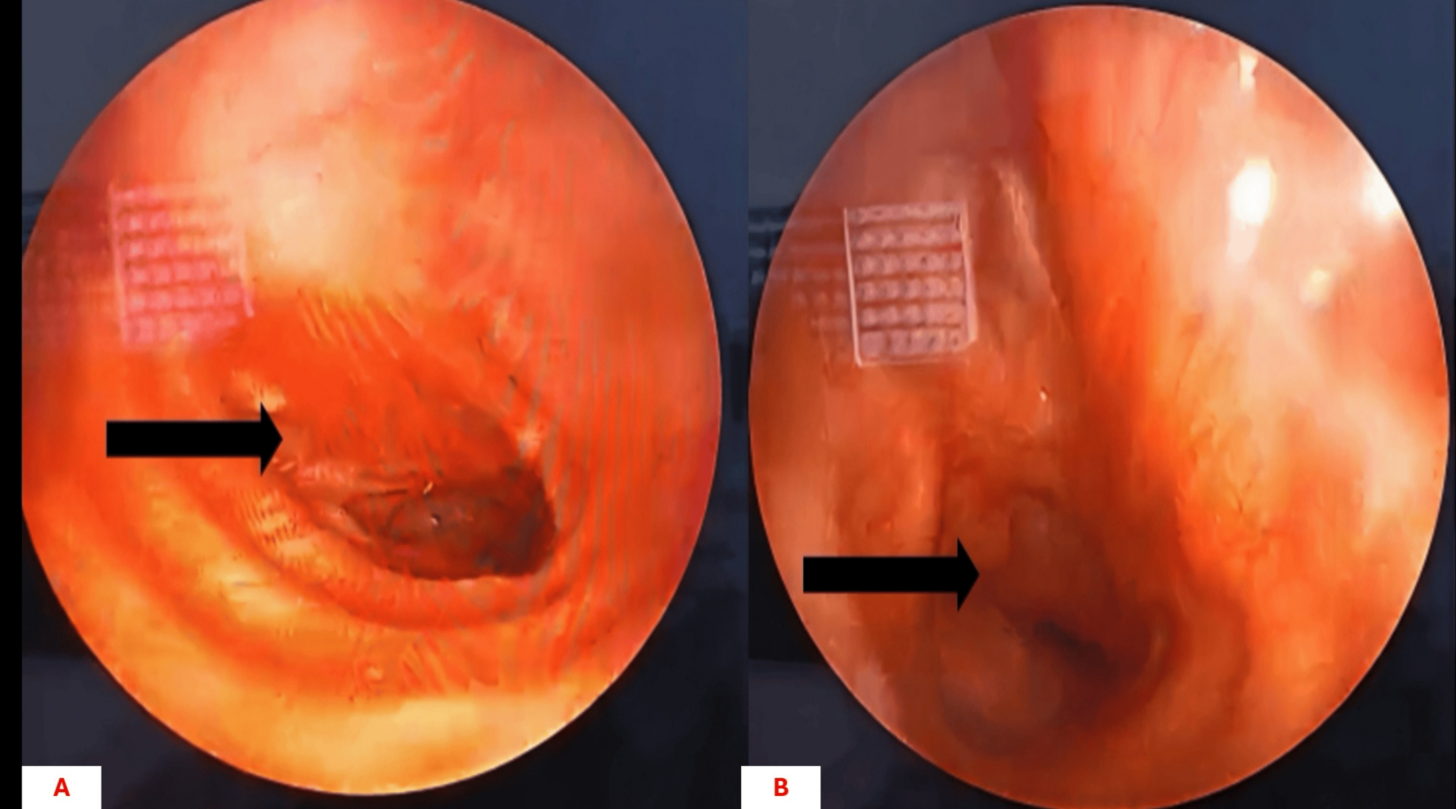
Follow-up bronchoscopic examination. (A) Bronchoscopic view six months after surgery showing the bronchial stump (arrow) without evidence of recurrence. (B) Bronchoscopic image confirming the absence of residual or recurrent endobronchial tumor (arrow).

Considering the risk for late local recurrence and delayed distant metastasis, long-term multidisciplinary surveillance is essential. In this patient, a six-month follow-up demonstrated clinical asymptomatic status, with chest CT showing post-pneumonectomy changes without residual or recurrent tumor (Figures 6A-6B), and bronchoscopic evaluation confirmed the absence of tumor relapse (Figures 7A-7B). This short-term outcome supports the effectiveness of complete surgical resection, but continued long-term monitoring is warranted to detect potential late recurrence.

## Discussion

Airway ACC is a rare malignant tumor representing approximately 0.1%-0.2% of primary lung tumors. This tumor was originally described by Theodor Billroth and arises from salivary gland-type structures distributed along the tracheobronchial tree [1].

ACC typically affects young adults and is not strongly associated with tobacco exposure. The clinical presentation is usually insidious and dominated by symptoms related to progressive central airway obstruction. Due to the nonspecific nature of these manifestations, misdiagnosis or delayed diagnosis is common [2].

Thoracic computed tomography is the first-line imaging modality to assess tumor location, airway involvement, and secondary complications such as post-obstructive pneumonia or lung atelectasis. However, the radiological features of ACC are not pathognomonic, and histopathological confirmation remains mandatory [5].

Histologically, ACC is characterized by cribriform, tubular, or solid architectural patterns composed of epithelial and myoepithelial tumor cells. Immunohistochemical analysis typically demonstrates positivity for CK7, CD117, p63, and CK5/6, and negativity for TTF-1 and neuroendocrine markers [6].

Molecular alterations, particularly the MYB-NFIB gene fusion, have been identified in a subset of tumors and may represent a potential therapeutic target in advanced disease [7].

The extent of obstruction, tumor dimensions, and histopathologic features were analyzed to justify surgical choice and postoperative surveillance [8]. While parenchyma-sparing procedures are preferred in young patients with slow-growing tumors, the extent of airway involvement and proximity to the carina necessitated pneumonectomy with carinal resection in this case. Complete resection with negative margins remains the primary determinant of prognosis [9]. This case demonstrates the importance of collaborative planning between pulmonologists, thoracic surgeons, anesthesiologists, and pathologists to optimize outcomes in complex airway tumors.

The tumor is generally regarded as relatively resistant to conventional chemotherapy, whereas radiotherapy may be considered as adjuvant therapy after incomplete resection or in unresectable cases [10].

ACC is characterized by an indolent but persistent course, with a well-recognized tendency for late local recurrence and delayed distant metastasis, even after apparently curative surgery. Prognosis mainly depends on tumor stage, completeness of surgical resection, and the presence of metastatic disease. Therefore, long-term clinical and radiological surveillance is essential to ensure early detection of recurrence [11].

## Conclusions

This case highlights the diagnostic challenges, individualized surgical decision-making, and the importance of long-term follow-up in primary bronchial ACC.

Early recognition of persistent airway symptoms is essential to prevent delayed diagnosis and advanced airway obstruction. Surgical management should be tailored to the tumor extent and anatomical constraints, and pneumonectomy may be required when parenchyma-sparing bronchoplastic procedures are not feasible.

Histopathologic findings, particularly cribriform architecture and perineural invasion, provide important prognostic information and support the need for prolonged surveillance.

Overall, this case underscores that tumor biology and airway involvement remain key determinants of surgical strategy and long-term management, emphasizing the importance of a multidisciplinary approach in optimizing patient outcomes.
